# Removal of Positively Buoyant *Planktothrix rubescens* in Lake Restoration

**DOI:** 10.3390/toxins12110700

**Published:** 2020-11-05

**Authors:** Miquel Lürling, Maíra Mucci, Guido Waajen

**Affiliations:** 1 Aquatic Ecology and Water Quality Management Group, Department of Environmental Sciences, Wageningen University, Droevendaalsesteeg 3a, 6708 PB Wageningen, The Netherlands; maira.mucci@wur.nl; 2 Water Authority Brabantse Delta, Team Knowledge, P.O. Box 5520, 4801 DZ Breda, The Netherlands; g.waajen@brabantsedelta.nl

**Keywords:** in-lake measures, lake restoration, Floc and Lock, Kill, Floc and sink, Hydrogen peroxide, Phoslock, PAC

## Abstract

The combination of a low-dose coagulant (polyaluminium chloride—‘Floc’) and a ballast able to bind phosphate (lanthanum modified bentonite, LMB—‘Sink/Lock’) have been used successfully to manage cyanobacterial blooms and eutrophication. In a recent ‘Floc and Lock’ intervention in Lake de Kuil (the Netherlands), cyanobacterial chlorophyll-*a* was reduced by 90% but, surprisingly, after one week elevated cyanobacterial concentrations were observed again that faded away during following weeks. Hence, to better understand why and how to avoid an increase in cyanobacterial concentration, experiments with collected cyanobacteria from Lakes De Kuil and Rauwbraken were performed. We showed that the *Planktothrix rubescens* from Lake de Kuil could initially be precipitated using a coagulant and ballast but, after one day, most of the filaments resurfaced again, even using a higher ballast dose. By contrast, the *P. rubescens* from Lake Rauwbraken remained precipitated after the Floc and Sink/Lock treatment. We highlight the need to test selected measures for each lake as the same technique with similar species (*P. rubescens*) yielded different results. Moreover, we show that damaging the cells first with hydrogen peroxide before adding the coagulant and ballast (a ‘Kill, Floc and Lock/Sink’ approach) could be promising to keep *P. rubescens* precipitated.

## 1. Introduction

Eutrophication—the over-enrichment of surface waters with nutrients—is the largest water quality issue worldwide [1]. It may result in a massive proliferation of cyanobacteria in lakes, ponds, and reservoirs [2,3]. Inasmuch as several strains of the most abundant, cosmopolite cyanobacteria may produce potent toxins, cyanobacterial blooms may impair ecosystems services, such as drinking water production, irrigation, recreation, aquaculture, and fisheries [4]. Clearly, managing eutrophication and reducing cyanobacterial blooms is a significant priority, but ongoing anthropogenic activities and climate change are predicted to aggravate further eutrophication and cyanobacterial blooms [4,5,6,7,8,9]. The Organisation for Economic Cooperation and Development (OECD) is already referring to eutrophication and harmful blooms as “becoming a global epidemic” with annual costs associated with nutrient pollution in Australia, Europe, and the USA at over 100 billion USD [10]. Hence, more effort of authorities is needed to control eutrophication and cyanobacterial blooms.

The most logical management strategy to mitigate eutrophication is to reduce the external nutrient inputs to surface water (e.g., [11,12,13]). However, this is easier said than done. About 70% of the point source nutrient pollution from municipal and industrial wastewater is treated in well-developed countries, while this is only 10% in low-income countries [14]. In OECD countries, nowadays, eutrophication is largely caused by legacies from the past and diffuse nutrient pollution from mostly agricultural activities [10,15]. Nutrient legacies in lake beds have built up over many years and will keep on fueling cyanobacterial blooms for years or decades after a successful reduction of the external nutrient load [16,17,18]. Recovery can be speeded up by targeting the legacy phosphorus pool [19]. Non-point source diffuse-nutrient pollution is more difficult to tackle and requires catchment-wide measures that may come with time lags of decades to centuries before water quality improves [20,21]. Consequently, in-lake measures are needed to bring real-time relief from either targeting cyanobacteria directly or indirectly via a strong reduction in nutrient availability [22]. A whole range of in-lake measures is proposed, several of which are not effective at all, but effective ones include algaecides, coagulants, and phosphate binders [22].

The combination of a low-dose coagulant (polyaluminium chloride, PAC—‘Floc’) and a phosphate binder (lanthanum modified bentonite, LMB—‘Lock’) was applied successfully in the Dutch stratifying Lake Rauwbraken [23]. This ‘Floc and Lock’ intervention effectively aggregated a developing bloom of *Aphanizomenon flos-aquae*, sedimented the aggregates out of the water column, reduced water column phosphate, strongly lowered sediment phosphate release, and improved water quality for more than 10 years after the intervention [23,24,25]. Likewise, in Lake De Kuil (the Netherlands) a system analysis revealed that around 95% of the phosphorus loading was released from the sediment, while the lake was also suffering from an *A. flos-aquae* bloom, which made the water authority opt for a “Floc and Lock” intervention too [26]. A low dose of iron chloride (as coagulant—‘Floc’) was added together with LMB to the lake in 2009 and successfully reduced phosphorus and chlorophyll-*a* concentrations, hampered the P release from the lake bed, and improved water quality [26]. However, continuing diffuse P-inputs undermined the strongly improved water quality and led to a *Planktothrix rubescens* bloom in early 2017, 8 years after the intervention in 2009.

**Figure A1:**
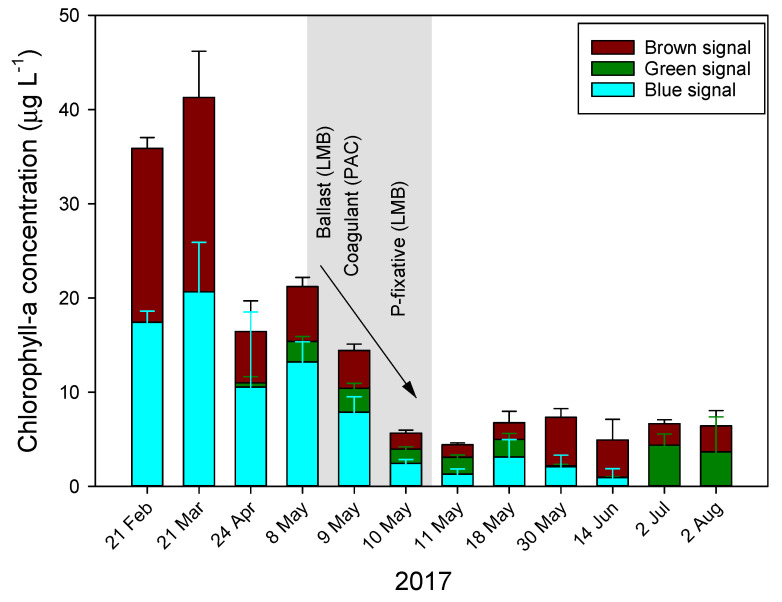
The course of water column (0–9 m) averaged chlorophyll-*a* concentrations (µg L^−1^) in Lake De Kuil in 2017 before, during (gray plane), and after a Floc and Lock intervention. Chlorophyll-a was determined with a PHYTO-PAM and separated in a blue-, green- and brown signal, based on different excitation wavelengths.

To counteract the developing cyanobacterial bloom and to prevent nuisance during the swimming season, the water authority decided to perform a second ‘Floc and Lock’ intervention. To this end, Lake De Kuil received a combined PAC and LMB treatment on 8–10 May 2017. At the moment of the application, the lake still experienced water column-dispersed *P. rubescens*. The combination of LMB as ballast and PAC as coagulant was added to clear the water column of these cyanobacteria, whilst injection of LMB in the hypolimnion would control phosphorus release from the sediment. On the first day (8 May), eight tons of LMB were added; on the second day, six tons of PAC (Calflock P-14) was added, both at the surface of the lake; and on the third day, the majority of the LMB (23 tons) was injected 4 m deeper in the water column. The cyanobacterial chlorophyll-*a*, which comprised the vast majority of the total chlorophyll-*a*, was reduced by almost 90%, but after one week, some elevated concentrations were observed that faded away in subsequent weeks (Appendix A; Figure A1) [27]. Because increasing cyanobacteria concentrations one week after the intervention (Figure A1) had not been observed in the previous studies [23,26], laboratory experiments were conducted with *P. rubescens* collected and concentrated from both Lake De Kuil and additional experiments included *P. rubescens* concentrated from Lake Rauwbraken. The experiments tested the hypothesis that some of the entrapped *P. rubescens* could escape sedimented flocs. Additional experiments focused on the possibility to include hydrogen peroxide to kill *P. rubescens* prior to ‘Floc and Lock’ and tested the hypothesis that a ‘Kill, Floc and Lock’ technique would not only effectively keep *P. rubescens* down, but also strongly reduce extracellular microcystin concentrations compared to solo hydrogen peroxide treatments.

## 2. Results

### 2.1. Floc and Sink Experiment—Ballast Dose

**Figure 1:**
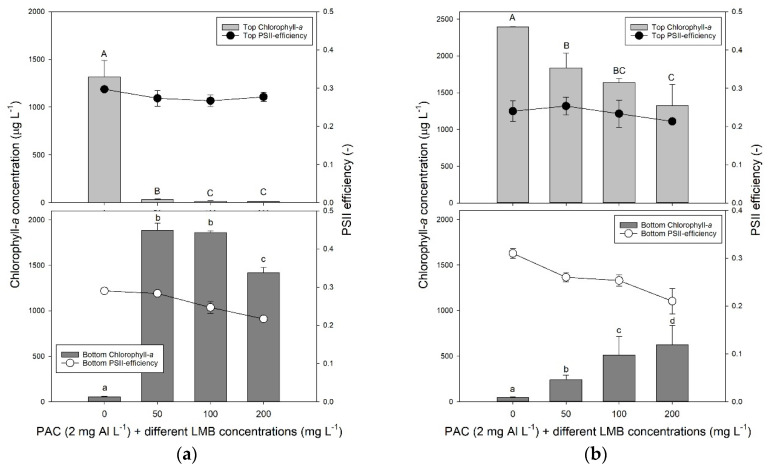
(**a**) Chlorophyll-a concentrations (μg L^−1^) in the top 2 mL (top light grey bars) and bottom 2 mL (lower dark grey bars) of 100 mL *P. rubescens* suspensions from De Kuil incubated for 1 h in the absence or presence of different concentrations ballast (50, 100, and 200 mg lanthanum modified bentonite (LMB) L^−1^) combined with the flocculent polyaluminium chloride (PAC) (2 mg Al L^−1^). Also included are the Photosystem II efficiencies (PSII) of the cyanobacteria collected at the surface of the tubes (filled circles) and at the bottom (open circles). Error bars indicate 1 standard deviation (SD, *n* = 3). Similar letters indicate homogeneous groups that are not different at the *p* < 0.05 level. (**b**) Similar to the panel (a), but now after 24 h incubation.

One hour after *P. rubescens* suspensions were treated with PAC (2 mg Al L^−1^) and different doses of ballast (50, 100 and 200 mg LMB L^−1^), in each treatment, the cyanobacteria were effectively translocated to the bottom of the tubes, while they accumulated in the top in the controls (Figure 1a). The one-way analysis of variance (ANOVA) indicated significant differences in chlorophyll-*a* concentrations at the water surface (*F*_3,11_ = 495.8; *p* < 0.001) and at the bottom (*F*_3,11_ = 835.1; *p* < 0.001) of the test tubes. In the control, the chlorophyll-*a* concentration at the water surface was significantly higher than in the treatments, while at the bottom, it was the opposite (Figure 1a). Hence, controls were significantly different from treatments. Photosystem II efficiencies (Φ_PSII_) in the top of the tube were similar (*F*_3,11_ = 2.40; *p* = 0.143) in controls and treatments; in the bottom they differed (*F*_3,11_ = 46.6; *p* < 0.001) and were significantly reduced in the 100 and 200 mg LMB L^−1^ treatments compared to control and 50 mg LMB L^−1^ treatment (Figure 1a). Notably, two hours later—first at the lowest LMB dose, and then followed by the 100 mg LMB L^−1^ treatments—settled flocks started to rise due to entrapped oxygen bubbles in the flocks (Appendix B).

After 24 h, most *P. rubescens* had surfaced again (Figure 1b; Appendix B). A one-way ANOVA indicated significant differences in chlorophyll-*a* concentrations at the water surface (*F*_3,11_ = 19.3; *p* < 0.001) and at the bottom (log-transformed data; *F*_3,11_ = 45.8; *p* < 0.001) of the test tubes. The LMB dose had a significant effect on the amount of chlorophyll surfacing and remaining at the bottom, with more chlorophyll accumulating at the lowest LMB dose and the least at the highest dose (Figure 1b). The Φ_PSII_ in the top of the tube were similar (*F*_3,11_ = 1.33; *p* = 0.330) in controls and treatments; in the bottom they differed (*F*_3,11_ = 19.5; *p* < 0.001) and were significantly reduced in the 100 and 200 mg LMB L^−1^ treatments compared to control and 50 mg LMB L^−1^ treatment (Figure 1b). The pH in the controls (pH = 8.38 ± 0.16) was significantly higher (*F*_3,11_ = 12.8; *p* = 0.002) than the pH in the three treatments, which were a pH of 8.10 (± 0.09) in the 50 mg LMB L^−1^ treatment, a pH of 7.98 (± 0.06) in the 100 mg LMB L^−1^ treatment, and of 7.93 (± 0.03) in the 200 mg LMB L^−1^ treatment.

**Figure A2:**
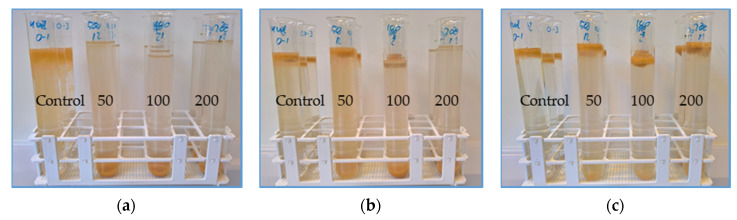
Pictures of test tubes with *P. rubescens* from lake De Kuil after one hour (**a**), three hours (**b**) and 24 h (**c**) exposure to 2 mg Al L^−1^ PAC + different doses of ballast; 50, 100 or 200 mg LMB L^−1^. Note that after three hours in the lower ballast doses, cyanobacteria started to float again. After 24 h, most have surfaced again.

It is obvious that, despite more cyanobacteria remaining precipitated with higher ballast dose, the majority of the settled flocks had risen again and accumulated at the water surface (Figure 1b and Figure A2).

### 2.2. Floc and Sink Experiment—Cyanobacteria Concentration

**Figure 2:**
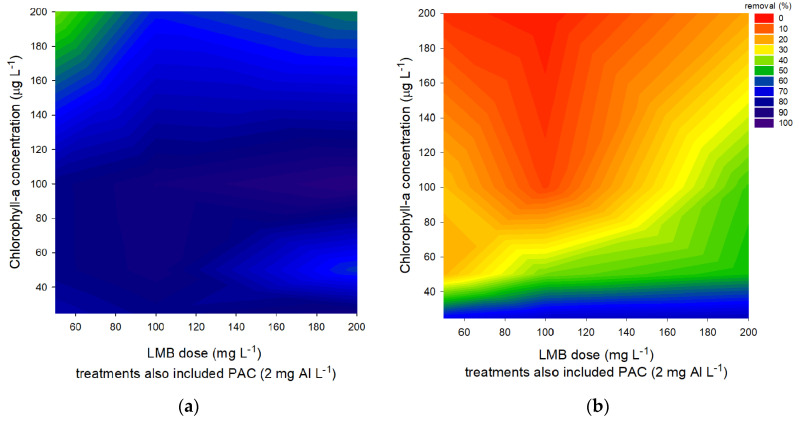
(**a**) Percentage of chlorophyll-*a* removal in *P. rubescens* suspensions with different chlorophyll-*a* concentrations (25–200 µg L^−1^) after 1 h exposure to different LMB concentrations mixed with the coagulant PAC (2 mg Al L^−1^). (**b**) Percentage of chlorophyll-*a* removal in *P. rubescens* suspensions with different chlorophyll-*a* concentrations (25–200 µg L^−1^) after 24 h exposure to different LMB concentrations mixed with the coagulant PAC (2 mg Al L^−1^).

A laboratory experiment using different concentrations of cyanobacteria and ballast was performed to examine how effective cyanobacterial biomass could be precipitated with PAC and LMB. The results after 1 h showed that 100 mg LMB L^−1^ sufficed to settle even the highest chlorophyll-*a* concentration used (200 μg chlorophyll L^−1^), while lower biomass could still be precipitated effectively using around 50 mg LMB L^−1^ (Figure 2a). However, the results after 24 h showed much lower removal and strongly hampered efficiency; even at low biomass, high amounts of ballast could only maximally remove 76% of the chlorophyll-*a* concentration (Figure 2b).

### 2.3. Effect of Hydrogen Peroxide on P. rubescens

The sensitivity of *P. rubescens* from Lake De Kuil to hydrogen peroxide was studied to test a possible strategy of killing/damaging *P. rubescens* before sweeping the water column clear of cyanobacteria with a coagulant and ballast. For comparison also, *P. rubescens* from Lake Rauwbraken was included.

**Figure 3:**
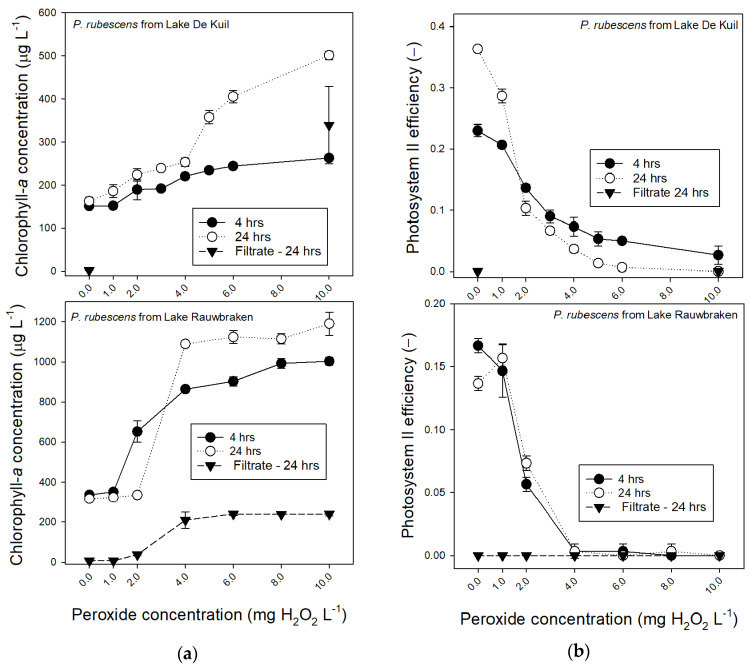
(**a**) Chlorophyll-a concentrations (μg L^−1^) in 25 mL *P. rubescens* suspensions from Lake De Kuil (top panel) and Lake Rauwbraken (bottom panel) after 4 h exposure (filled circles) and 24 h exposure (open circles) to different concentrations hydrogen peroxide (0–10 mg L^−1^). Also included are the chlorophyll concentrations determined in 0.45 µm filtered samples after 24 h (triangles). Note that for Lake De Kuil these were only tested for the controls (0 mg L^−1^) and the 10 mg H_2_O_2_ L^−1^ treatment. Error bars indicate 1 SD (*n* = 3). (**b**) Photosystem II efficiencies of *P. rubescens* suspensions from Lake De Kuil (top panel) and Lake Rauwbraken (bottom panel) after 4 h exposure (filled circles) and 24 h exposure (open circles) to different concentrations hydrogen peroxide (0–10 mg L^−1^). Also included are the Photosystem II efficiencies determined in 0.45 µm filtered samples after 24 h (triangles). Error bars indicate 1 SD (*n* = 3).

*P. rubescens* from Lake De Kuil was less sensitive to hydrogen peroxide (H_2_O_2_) than *P. rubescens* from Lake Rauwbraken even though the latter was incubated at almost twice as high chlorophyll-*a* concentrations (Figure 3a). The Photosystem II efficiency (Φ_PSII_) of *P. rubescens* from Lake Rauwbraken dropped to zero at 4 mg H_2_O_2_ L^−1^, while this was reached at 10 mg H_2_O_2_ L^−1^ for *P. rubescens* from Lake De Kuil (Figure 3b). Nonetheless, Φ_PSII_ had dropped strongly from 2 mg H_2_O_2_ L^−1^ and higher for both, reflecting damages to the cells. Strongly increased chlorophyll-*a* concentrations further exemplify this damage. As evidenced by filtrate measurements, this was caused partly by dissolved fluorescent pigments (Figure 3a), which did not yield any Φ_PSII_ (Figure 3b).

### 2.4. Efficacy of a Combined Hydrogen Peroxide and Floc and Sink Treatment on P. rubescens

**Figure 4:**
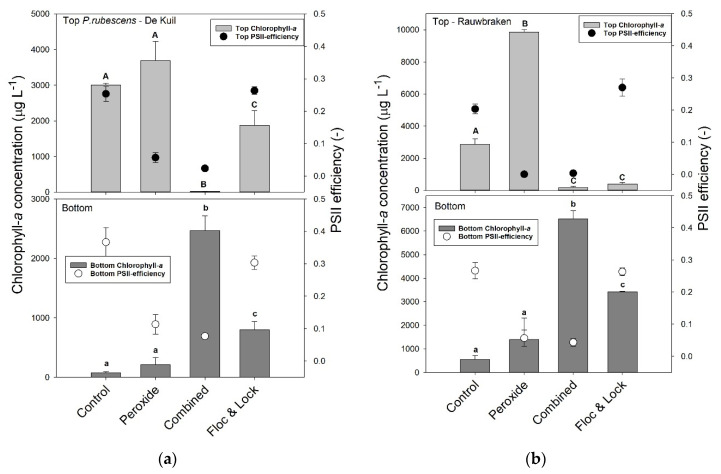
Chlorophyll-a concentrations (μg L^−1^) in the top 2 mL (top light grey bars) and bottom 2 mL (lower dark grey bars) of 100 mL cyanobacteria suspension from (**a**) Lake De Kuil and (**b**) Lake Rauwbraken after 24 h exposure to hydrogen peroxide (5 mg L^−1^), peroxide + coagulant (2 mg Al L^−1^) and ballast (200 mg LMB L^−1^) (combined) or only coagulant (2 mg Al L^−1^) and LMB (Floc & Lock). Also included are the Photosystem II efficiencies (PSII) of the cyanobacteria collected at the water surface (filled circles) and at the bottom (open circles). Error bars indicate 1 SD (*n* = 3). Similar letters indicate homogeneous groups that are not different at the *p* < 0.05 level.

In the series with *P. rubescens* from Lake De Kuil, the chlorophyll-*a* concentrations in the top of the test tubes were significantly different (*F*_3,11_ = 149.5; *p* < 0.001) between controls and the various treatments after 24 h incubation (Figure 4a). In the control and sole peroxide treatment (5 mg H_2_O_2_ L^−1^), chlorophyll-*a* concentrations were highest and not different from each other; in the Floc and Lock treatment (PAC, 2 mg Al L^−1^ and LMB, 200 mg L^−1^), chlorophyll-*a* concentrations were significantly lower, but still 100 x higher than in the combined treatment (H_2_O_2_, 5 mg L^−1^, PAC, 2 mg Al L^−1^ and LMB, 200 mg L^−1^) in which virtually all chlorophyll-*a* had remained at the bottom of the test tubes (Figure 4a). In the bottom of the tubes, chlorophyll-*a* concentrations were lowest in the control and sole peroxide treatment, significantly higher (log-transformed data; *F*_3,11_ = 401.8; *p* < 0.001) in the Floc and Lock treatment, and highest in the combined treatment (Figure 4a). The Φ_PSII_ in the top of the test tubes was significantly different among treatments (*F*_3,11_ = 206.9; *p* < 0.001), as was Φ_PSII_ in the bottom samples (*F*_3,11_ = 70.2; *p* < 0.001). The Holm–Sidak post hoc pairwise comparison revealed for both top and bottom water samples two homogenous groups: (1) the controls and the Floc and Lock treatments, and (2) both peroxide treatments (solo and combined).

In the series with *P. rubescens* from Lake Rauwbraken in both the controls and the sole peroxide treatments, the vast majority of the filaments aggregated at the water surface (Figure 4b). In contrast, in both the Floc and Lock treatment and the combined treatment, most cyanobacteria were at the bottom of the tube (Figure 4b). One-way ANOVA indicated significant differences in chlorophyll-*a* concentrations at the water surface (*F*_3,11_ = 1428; *p* < 0.001) and at the bottom (*F*_3,11_ = 86.4; *p* < 0.001) of the test tubes. The Φ_PSII_ in the top of the test tubes was significantly different among treatments (*F*_3,11_ = 237.7; *p* < 0.001), where Holm–Sidak post hoc pairwise comparison revealed that Φ_PSII_ in both peroxide treatments (sole and combined) were significantly lower than in control and the Floc and Lock treatment (Figure 4b). The Φ_PSII_ in the bottom of the test tubes was also significantly different among treatments (*F*_3,11_ = 120.8; *p* < 0.001), and Holm–Sidak post hoc pairwise comparison revealed two homogenous groups: 1) the controls and Floc and Lock treatments, and 2) both peroxide treatments.

**Figure 5:**
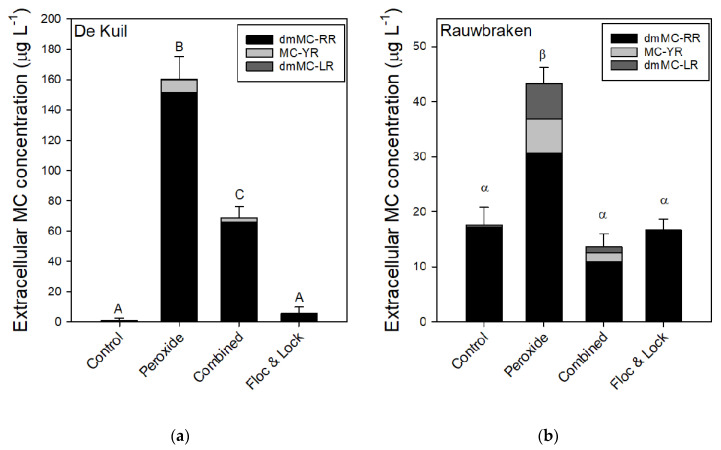
Extracellular microcystin (MC) concentrations (μg L^−1^) of three MC variants quantified in samples from *P. rubescens* suspensions from (**a**) Lake De Kuil and (**b**) Lake Rauwbraken after 24 h exposure to hydrogen peroxide (5 mg L^−1^), peroxide + coagulant (2 mg Al L^−1^) and ballast (200 mg LMB L^−1^) (combined) or only coagulant (2 mg Al L^−1^) and LMB (Floc & Lock). Error bars indicate 1 SD (*n* = 3). Similar letters indicate homogeneous groups that are not different at the *p* < 0.05 level.

The exposure of *P. rubescens* suspensions collected from Lake De Kuil to hydrogen peroxide caused a sharp increase in the concentration of extracellular microcystins (MCs) of which the variant dmMC-RR was most abundant (Figure 5a). The one-way ANOVA indicated significant differences (*F*_3,11_ = 226.1; *p* < 0.001); extracellular MC was lowest in control and Floc and Lock treatments and the highest in the solely peroxide treatment (Figure 5a). In the combined treatment, extracellular MC concentration was about 60% lower than in the sole peroxide treatment (Figure 5a). In suspensions with *P. rubescens* from Lake Rauwbraken only exposure to solely hydrogen peroxide caused significantly (*F*_3,11_ = 78.0; *p* < 0.001) elevated extracellular MC concentrations (Figure 5b).

## 3. Discussion

The experiments provided clear evidence that *P. rubescens* from Lake De Kuil could initially be precipitated using a coagulant and a ballast, but that after 24 h, most filaments had resurfaced again. Those results should be a warning when it comes to the use of short-term (1–2 h) tests to determine the efficacy of a coagulant and ballast in so-called ‘Floc and Sink’ assays [28,29,30,31]. The reason for this study was based on field observations that showed reoccurring *P. rubescens* after a ‘Floc and Lock’ treatment of Lake De Kuil in May 2017 [27]. However, in other whole lake ‘Floc and Lock’ interventions [23], including one in Lake De Kuil in 2009 [26], no such reappearance had been observed. In those lakes, another cyanobacterium (*Aphanizomenon flas-aquae*) was dominating at the time of intervention. Likewise, in an experiment in which sediment cores and over-standing water infested with *Microcystis aeruginosa* were treated with PAC + LMB, chlorophyll-*a* concentrations were within 1.5 h more than 90% lower than in the control, which remained low during the entire 13 days of the experiment [32]. Clearly, the outcome of those experiments in which the entrapped cyanobacteria stay alive is influenced by species/strain-specific characteristics.

One important feature of *Planktothrix* as a member of the Oscillatoriales is its motility, which is a gliding movement or positive phototactic orientation; an oriented movement towards light [33]. This movement could allow the filaments to crawl out of flocs, as flocs are composed of aggregates with differently sized pores [34]. Another characteristic of *P. rubescens* is that it is highly adapted to low light conditions and can even grow using low amounts of green light prevailing at depth [35,36]. Considering the relative shallowness of Lake De Kuil (maximum water depth ~9 m, average depth ~4 m), ongoing photosynthesis on the sediment with a cleared water column would have been very likely. Consequently, flotation of flocs by oxygen bubbles generated by photosynthesis may occur [37], which could lead to resurfacing of some of the flocs.

Despite the fact that higher ballast doses kept more cyanobacteria at the bottom of the tubes (see Figure 1b), even a ballast dose of 200 mg LMB L^−1^ was insufficient to keep most of the *Planktothrix* at the bottom of the test tubes. In line with previous findings [29], more ballast was needed to remove higher cyanobacterial biomass, but only low cyanobacterial biomass could be kept at the bottom of the tubes for at least 24 h (see Figure 2b). During the application in Lake de Kuil, the biomass in the first 4 m was around 24 μg chlorophyll L^−1,^ and around 30 mg LMB L^−1^ (based on the whole lake volume was applied at the surface as ballast before the PAC application [27]. This means the ballast dose has been higher than 30 mg LMB L^−1^ in the upper one–two meters of the water column. The results of our experiments suggested that at 25 μg chlorophyll L^−1^, when 50 mg LMB L^−1^ was added, the removal efficiency was maximally 68%. Extrapolating this to the field implies that even when the cyanobacterial biomass had been reduced by two-thirds over time, the reoccurring biomass is large enough to accumulate in relatively high densities at the shore. Hence, biomass plays a role in determining the amount of ballast needed to remove the cells efficiently. However, in this case even using the highest amount of ballast at the lowest chlorophyll-*a* concentrations could not prevent a return to the water column after 24 h. Adding even more ballast would probably not have kept all the biomass at the bottom for reasons of motility and ongoing photosynthesis.

Consequently, additional measures to kill or damage the cyanobacteria and then remove them from the water column seem a strategy to control the nuisance. Such “Kill, Floc and Sink” combination [25] has already been tested with hydrogen peroxide as cyanobacteriocide combined with the coagulant polymeric ferric sulphate (PFS) and lake sediment [38]. In a 91 m^2^ enclosure, a *Microcystis* bloom was treated with 60 mg H_2_O_2_ L^−1^, followed 2 h later by combined 20 mg PFS L^−1^ and 2 g sediment L^−1^ as ballast [38]. Because effective hydrogen peroxide doses against *Planktothrix* sp. (e.g., [39,40,41,42]), are much lower than the high concentration used by Wang et al. [38], hydrogen peroxide was tested in a lower dosage range, which also implies limited side effects on non-target organisms [39,43]. Inasmuch as sensitivity to hydrogen peroxide might differ between cyanobacteria [42] and between strains [44], the sensitivity to hydrogen peroxide of *P. rubescens* from Lake De kuil was compared to that of *P. rubescens* concentrated from Lake Rauwbraken. Photosystem II efficiency (Φ_PSII_) was chosen as an endpoint because it reflects the fitness of photosynthetic organisms and can be used to demonstrate the damage of H_2_O_2_ to the photosystem of cyanobacteria [45,46,47]. The *P. rubescens* from Lake Rauwbraken was more sensitive than *P. rubescens* from Lake De Kuil as its Φ_PSII_ was already zero at 4 mg H_2_O_2_ L^−1^, while Φ_PSII_ of *P. rubescens* from Lake De Kuil dropped to zero at 10 mg H_2_O_2_ L^−1^, but a strong decline was already observed at much lower concentrations of 2 mg L^−1^, which is comparable to findings with other cyanobacteria [39,46].

At these H_2_O_2_ concentrations, the chlorophyll-*a* concentrations (in μg L^−1^) determined by the PHYTO-PAM were also elevated. This is caused by the detachment of pigments from the thylakoid membranes [46] and leakage of them into the water. Those water soluble extracellular pigments from cyanobacteria can contribute considerably to the detected fluorescence signal, which does not reflect an increase of biomass [48]. The increase in the filterable chlorophyll-*a* without any Φ_PSII_ is a clear indicator of this cell leakage as in general the release of intracellular components is an indication of membrane damage [49]. Given that these extracellular pigments were still elevated 24 h after application, the oxidizing power of the introduced H_2_O_2_ was not enough to destroy released cell constituents. Likewise, in the combined hydrogen peroxide and Floc and Sink experiment, the H_2_O_2_ treatments had higher chlorophyll-*a* concentrations than their corresponding controls (the water surface for sole peroxide and control; see Figure 4).

In the combined hydrogen peroxide and Floc and Sink experiment, we chose a dose of 5 mg H_2_O_2_ L^−1^, which was sufficient to damage *P. rubescens* from Lake De Kuil for a period of three hours after which the coagulant and ballast were added. This was sufficient to reduce the viability of the filaments to such an extent that they remained precipitated after 24 h incubation. The chlorophyll-*a* concentration in the top of Lake De Kuil tubes was 0.8 (± 0.3) % of that in the bottom, while in the Lake Raubraken combined treated tubes, it was 2.8 (± 1.4) %. When only coagulant and ballast were used, a considerable part of the *P. rubescens* from Lake De Kuil had resurfaced after 24 h, just as observed in the previous experiments. In contrast, *P. rubescens* from Lake Rauwbraken remained precipitated after a sole Floc and Sink treatment. In the Lake Rauwbraken series the chlorophyll-*a* concentration in the top of the sole Floc and Sink tubes was 11 (± 3) % of that in the bottom, while in the Lake De Kuil series, it was 294 (± 96) %. Evidently, the preceding H_2_O_2_ treatment was effective in keeping *P. rubescens* at the bottom of the tubes. A side effect of the H_2_O_2_ treatment was leakage of cell constituents, such as pigments and toxins (microcystins, MC). In both *P. rubescens*, exposure to H_2_O_2_ led to strongly elevated extracellular MC concentrations, which has also been observed for *Microcystis aeruginosa* exposed to H_2_O_2_ [50]. However, when followed by coagulant and ballast, the extracellular MC concentrations were strongly reduced and for Lake Rauwbraken, even similar to the controls. These results match with the recently reported capacity of LMB to lower dissolved MC concentrations; LMB dosed at 50, 100, and 150 ppm decreased MC concentrations by 61.2%, 86.0%, and 75.4% relative the controls, respectively [51]. However, in the sole Floc and Sink treatments, no further reduction of extracellular MC concentrations was observed. Inasmuch as a Floc and Sink treatment will not damage filaments or cells, and thus will not liberate MCs rapidly, its strongest effect on MC concentration will be via precipitation of cyanobacteria and thereby the removal of particulate MCs from the water column. Nonetheless, concomitant reduction of extracellular MCs is possible, as was shown by a combined chitosan-nano scale montmorillonite treatment that effectively precipitated *Microcystis aeruginosa* (94% removal) and removed 90% of the extracellular MCs within one hour [52]. Clearly, concomitant measurements of cyanobacterial biomass and cyanotoxin concentrations during an intervention are strongly recommended.

The effective precipitation of *P. rubescens* using a combined hydrogen peroxide and Floc and Lock treatment also indicates that despite the fact that H_2_O_2_ will decay and produce oxygen, this is not leading to the surfacing of flocs. Wang et al. [38] reported that “the floc of *Microcystis* bloom was oxygen-rich…”, but also that the flocs were deposited on the sediment. However, when calcium peroxide was used as cyanobacteriocide combined with chitosan as a coagulant and red soil as ballast, part of the settled cyanobacteria/ballast flocs migrated upwards again [28]. Hence, the separation of the oxidizing agent (added 3 h before the coagulant and ballast) prevents entrapment of oxygen bubbles inside the aggregates formed; and seems more effective than including a granular formulation together with the coagulant and ballast.

Our experiments yielded insight that a combined hydrogen peroxide and ‘Floc and Lock’ treatment could be effective in keeping *P. rubescens* precipitated and showed that similar species (*P. rubescens*), but from two different lakes, yielded dissimilar results. This further underpins the necessity to test selected measures for each lake first. Not a single lake is unique, and this also holds for the target organisms, even when belonging to the same species.

## 4. Conclusions

Short-term (1–2 h) tests to determine the efficacy of precipitation of cyanobacteria by a coagulant and ballast in so-called ‘Floc and Sink’ assays should be extended to at least 24 h. Motile or low-light adapted cyanobacteria, such as *P. rubescens*, may cause resurfacing of initially settled flocs within 24 h. Using hydrogen peroxide preceding the ‘Floc and Sink’ treatment seems effective in keeping the cyanobacteria precipitated and thus out of the water column. Moreover, the coagulant and ballast reduce extracellular MCs liberated from damaging the cyanobacteria by H_2_O_2_. Up-scaled experiments are needed to test the proposed “Kill, Floc and Sink/Lock” approach under more realistic (field) conditions prior to field applications.

## 5. Materials and Methods

On 30 May 2017, samples were taken from Lake De Kuil (the Netherlands). The lake had orange-colored, odorous surface scums accumulated in some shore regions. Microscopy revealed it consisted of *Planktothrix rubescens*. A large volume (10 L) surface accumulated material was collected to have some higher biomass samples to be used in the experiments. Water samples over the vertical, as well as samples from different sites were collected. In the laboratory, accumulated material and collected water were mixed to create suspensions that were used in experiments to test combined treatments of a coagulant (Floc) and a ballast (Sink) as well as treatments that include hydrogen peroxide (Kill).

### 5.1. Floc and Sink Experiment—Ballast Dose

The total dose of PAC applied to Lake De Kuil was 6 tons of Calflock P-14 (Caldic Belgium N.V., Hemiksem, Belgium), which contains 7.2% Al, and has a specific gravity of 1.31 kg L^−1^. An average volume of 268,000 m^3^ yielded a PAC dose of 2.1 mg Al L^−1^. The lanthanum modified bentonite (LMB) Phoslock^®^ (Phoslock Europe Ltd., Manchester, U.K.) was dosed at 31 tons yielding around 116 mg L^−1^. The LMB ballast, however, will surely have been less as only part of the LMB had been added the first day. Therefore, the effect of different ballast doses on the efficiency of *P. rubescens* removal was studied. The PAC dose was kept at 2 mg Al L^−1^ (Floc), while LMB as ballast (Sink) was dosed at 50, 100, and 200 mg L^−1^. The experiment was conducted in 125 mL glass tubes filled with 100 mL of a *P. rubescens* suspension created by mixing surface accumulated *P. rubescens* and water collected from Lake De Kuil. This *P. rubescens* infested test water had a chlorophyll-*a* concentration of 114 (± 5) µg L^−1^. Treatments were run in triplicate, while three non-treated tubes served as controls. A slurry of LMB was added to the treatments, immediately followed by the addition of PAC. All suspensions—controls and treatments—were stirred using a metal rod and left untouched for one hour. Then, 2 mL samples were taken from both the top as well as the bottom of each tube using a 10 mL Eppendorf Varipette pipette (Eppendorf Nederland B.V., Nijmegen, the Netherlands). These samples were analyzed on their chlorophyll-*a* concentrations and Photosystem II efficiencies (Φ_PSII_) using a PHYTO-PAM phytoplankton analyzer (HeinzWalz GmbH, Effeltrich, Germany). After measuring, the samples were gently placed back in the region from where they were taken. After 24 h, again, 2 mL samples were taken from both the top as well as the bottom of each tube and measured as indicated before. The pH was measured using a WTW multi 340i meter (WTW GmbH and Co. KG, Weilheim, Germany).

The chlorophyll-*a* concentrations after 1 h and after 24 h in the top of the test tubes and in the bottom were evaluated running separate one-way ANOVAs using the toolpack SigmaPlot version 14.0 (Systat Software Inc., San Jose, CA, USA, 2017). The same was done for the Φ_PSII_ and for the pH. Significant differences were distinguished using Holm–Sidak post-hoc pairwise comparisons. Normality (normality test: Shapiro-Wilk) and homogeneity of variance (equal variance test: Brown–Forsythe) were checked prior to running the one-way ANOVAs.

### 5.2. Floc and Sink Experiment—Cyanobacteria Concentration

A second ‘Floc and Sink’ experiment was performed to test the hypothesis that the cyanobacterial biomass was too high to be removed. To this end, different combinations of cyanobacteria and ballast concentrations were tested: LMB (50, 100 and 200 mg L^−1^) in combination with PAC (2 mg Al L^−1^) were added to various cyanobacteria concentrations (25, 50, 100 and 200 μg chlorophyll-*a* L^−1^) that were determined by PHYTO-PAM. One tube per cyanobacteria concentration was left untreated. After 1 h, samples from top and bottom were taken. In addition, an extra series was left standing for 24 h and measured then as described above. Hence, this experiment comprised 4 chlorophyll-*a* concentrations × 4 LMB doses × 2 series = 32 experimental tubes.

### 5.3. Effect of Hydrogen Peroxide on P. rubescens

The Floc and Sink experiments yielded insight that additional measures to kill/damage *P. rubescens* prior to adding a coagulant and ballast seem a possible strategy to control the nuisance. Hereto, the sensitivity of *P. rubescens* to hydrogen peroxide was tested. For comparison also *P. rubescens* from Lake Rauwbraken was included, which was sampled using a plankton net from deeper water layers where a low biomass resided [53]. *P. rubescens* from Lake Rauwbraken was further concentrated by pipetting off the surface-accumulated material and transferring it into filtered lake water. The material was visually inspected under the light microscope, no other cyanobacteria were observed.

Aliquots of 25 mL *P. rubescens* suspensions from Lake Rauwbraken (318 ± 34 μg chlorophyll-*a* L^−1^; Φ_PSII_ = 0.31 ± 0.01) and from Lake De Kuil (318 ± 34 μg chlorophyll-*a* L^−1^; Φ_PSII_ = 0.30 ± 0.01) were transferred into transparent polystyrene vials (VWR^®^ vials with cap, VWR International B.V., Amsterdam, the Netherlands). Hydrogen peroxide (H_2_O_2_ 30%, 1.07209.0500, Merck KGaA, Darmstadt, Germany) was tested in triplicate concentrations of 0, 1, 2, 4, 6, 8 and 10 mg L^−1^ for Lake Rauwbraken and at 0, 1, 2, 3, 4, 5, 6, and 10 mg L^−1^ for Lake De Kuil. It was pipetted from a 100× diluted stock, where after the vials were closed with a lid and gently shaken. After 4 h, the vials were shaken, a 2 mL subsample analyzed on their chlorophyll-*a* concentrations and Photosystem II efficiencies (Φ_PSII_) using the PHYTO-PAM phytoplankton analyzer and pipetted back into the vial. After 24 h, the measurement was repeated.

### 5.4. Efficacy of a Combined Hydrogen Peroxide and Floc and Sink Treatment on P. rubescens

The efficiency of a combined H_2_O_2_, PAC and LMB treatment (‘Kill, Floc and Sink’) on removing *P. rubescens* from the water column was examined. This experiment tested the hypothesis that H_2_O_2_ would damage *P. rubescens* cells enough to strongly hamper photosynthesis and buoyancy, which would keep filaments aggregated in flocs at the bottom of the test units. Based on the peroxide exposure experiment (described in Section 5.3) a working dose of 5 mg H_2_O_2_ L^−1^ was chosen. Aliquots of 100 mL concentrated *P. rubescens* from Lake Rauwbraken (202 ± 5 μg chlorophyll-*a* L^−1^; Φ_PSII_ = 0.35 ± 0.03) was transferred to 12 glass tubes of 125 mL. Similarly, samples of 100 mL from Lake De Kuil concentrate (156 ± 4 μg chlorophyll-*a* L^−1^; Φ_PSII_ = 0.32 ± 0.03) was brought into 12 other tubes. Three tubes of each series remained untreated (controls), six tubes of each series were treated with peroxide (5 mg H_2_O_2_ L^−1^). After three hours, three of the peroxide treated tubes were treated with a coagulant (PAC, 2 mg Al L^−1^) and ballast (LMB, 200 mg L^−1^), while the remaining three tubes per series were treated with an only coagulant (PAC, 2 mg Al L^−1^) and ballast (LMB, 200 mg L^−1^), which is referred to as a Floc and Lock treatment. The tubes were incubated for 24 h in the laboratory, where after 2 mL samples from the top of the test tubes and from the bottom of each tube were collected. These samples were analyzed on their chlorophyll-*a* concentrations and the Φ_PSII_.

From the middle of each tube, a 5 mL sample was taken with a syringe and filtered through a 0.45 μm unit filter (Aqua 30/0.45CA, Whatman, Germany). The filtrates were collected in 8 mL glass tubes and evaporated to dryness in a Speedvac (Thermo Scientific Savant SPD121P, Waltham, MA, USA). The dried filtrates were reconstituted with 800 μL methanol and transferred to 2-mL Eppendorf vials with a cellulose-acetate filter (0.2 μm, Grace Davison Discovery Sciences, Deerfield, IL, USA) and centrifuged for 5 min at 16,000× *g* (VWR Galaxy 16DH, VWR International, Buffalo Grove, IL, USA). Filtrates were transferred to amber glass vials and analyzed for eight microcystins (MC) variants (dm-7-MC-RR, MC-RR, MC-YR, dm-7-MC-LR, MC-LR, MC-LY, MC-LW, and MC-LF) and nodularin (NOD) by LC-MS/MS. The variants dm-7-MC-RR, MC-YR, dm-7-MC-LR, MC-LR, MC-LY and NOD were obtained from DHI Lab products (Hørsholm, Denmark), the variants MC-RR, MC-LF and MC-LW were obtained from Novakits (Nantes, France). The LC-MS/MS was performed as described in Lürling and Faassen [54]. The concentration of each MC variant in the samples was calculated against a calibration curve of each standard and subsequently corrected for recovery, which had been determined for each variant by spiking a cyanobacterial matrix [54].

The chlorophyll-*a* concentrations in the top of the test tubes and at the bottom were evaluated by running separate one-way ANOVAs using the toolpack SigmaPlot version 14.0. Likewise, the Φ_PSII_ in the top of the test tubes and the bottom were evaluated by running separate one-way ANOVAs. Also the total MC concentrations were evaluated with a one-way ANOVA. Holm–Sidak post-hoc pairwise comparisons tests were run to distinguish significant differences. Normality (normality test: Shapiro–Wilk) and homogeneity of variance (equal variance test: Brown–Forsythe) was checked before running the one-way ANOVAs.

## Appendix A

The Floc and Lock intervention in Lake De Kuil (8–10 May 2017) effectively precipitated a bloom of the cyanobacterium *P. rubescens*. Total chlorophyll-*a* concentration was reduced by 80% and the blue signal—indicative for phycocyanin-containing cyanobacteria—was reduced by more than 90% (Figure A1). The PHYTO-PAM uses four different excitation wavelengths, provided by light-emitting diodes (LEDs) peaking at 470, 535, 620, and 659 nm that allow a separation between cyanobacteria, green algae and diatoms/dinoflagellates [55]. However, the cyanobacteria that can be distinguished are those containing mostly phycocyanin, while phycoerythrin-containing species, such as *P. rubescens*, will not be accurately placed in the “blue channel”. A LED with a wavelength around 570 nm would be needed for the excitation of phycoerythrin, which would allow identification of phycoerythrin containing cyanobacteria and cryptophyta [56]. Hence, it is likely part of the signal picked up in the “brown channel” originated from *P. rubescens.* Nonetheless, the intervention reduced chlorophyll-*a* concentrations strongly, but after one week (18 May) a slight increase in the blue channel was detected (Figure A1).

## Appendix B

*P. rubescens* could initially be settled to the bottom of the test tubes rise due to entrapped oxygen bubbles in the flocks (see pictures below).
